# Characteristics of Adults Hospitalized for a Major Depressive Disorder: Results from the Multicenter OASIS-D Study

**DOI:** 10.1192/j.eurpsy.2023.755

**Published:** 2023-07-19

**Authors:** C. U. Correll, F. Bermpohl, N. Schoofs, R. Bathe-Peters, K. Pfeifer, P. Falkai, C. Schüle, F. Pan-Montojo, E. Y. M. Wang, A. Reif, C. Reif-Leonhard, S. Schillo, P. Getty, M. Adli, R. Papenfuß, F. Jessen, F. Salimi-Dafsari, M. Bauer, U. Lewitzka, C. Otte, L. Graumann, D. Piber, S. Weyn-Banningh, A. Meyer-Lindenberg, A. Böhringer, F. Heuer, V. B. Nöhles

**Affiliations:** 1 Child and Adolescent Psychiatry, Charite Universitaetsmedizin, Campus Virchow-Klinikum; 2 Department of Psychiatry and Psychotherapy, Charite Universitaetsmedizin, Campus St. Hedwig Hospital; 3 Department of Psychiatry and Psychotherapy, Charite Universitaetsmedizin, Campus St. Hedwig Hospital, Berlin; 4 Clinic for Psychiatry and Psychotherapy, Ludwig-Maximilians-University Munich, Munich; 5 Department of Psychiatry, Psychosomatic Medicine and Psychotherapy, University Hospital Frankfurt, Frankfurt am Main; 6 Department of Psychiatry and Psychotherapy, Charite Universitaetsmedizin, Campus Mitte, Berlin; 7 Department of Psychiatry and Psychotherapy, University of Cologne, Cologne; 8 Department of Psychiatry and Psychotherapy, University Hospital Carl Gustav Carus, Dresden; 9 Department of Psychiatry and Psychotherapy, Charite Universitaetsmedizin, Campus Benjamin Franklin, Berlin; 10 Department of Psychiatry and Psychotherapy, Central Institute of Mental Health, Mannheim, Germany

## Abstract

**Introduction:**

Major Depressive Disorder (MDD) is one of the most common mental illnesses worldwide and is strongly associated with suicidality. Commonly used treatments for MDD with suicidality include crisis intervention, oral antidepressants (although risk of suicidal behavior is high among non-responders and during the first 10-14 days of the treatment) benzodiazepines and lithium. Although several interventions addressing suicidality exist, only few studies have characterized in detail patients with MDD and suicidality, including treatment, clinical course and outcomes. Patient Characteristics, Validity of Clinical Diagnoses and Outcomes Associated with Suicidality in Inpatients with Symptoms of Depression (OASIS-D)-study is an investigator-initiated trial funded by Janssen-Cilag GmbH.

**Objectives:**

For population 1 out of 3 OASIS-D populations, to assess the sub-population of patients with suicidality and its correlates in hospitalized individuals with MDD.

**Methods:**

The ongoing OASIS-D study consecutively examines hospitalized patients at 8 German psychiatric university hospitals treated as part of routine clinical care. A sub-group of patients with persistent suicidality after >48 hours post-hospitalization are assessed in detail and a sub-group of those are followed for 6 months to assess course and treatment of suicidality associated with MDD. The present analysis focuses on a preplanned interim analysis of the overall hospitalized population with MDD.

**Results:**

Of 2,049 inpatients (age=42.5±15.9 years, females=53.2%), 68.0% had severe MDD without psychosis and 21.2% had moderately severe MDD, with 16.7% having treatment-resistant MDD. Most inpatients referred themselves (49.4%), followed by referrals by outpatient care providers (14.6%), inpatient care providers (9.0%), family/friends (8.5%), and ambulance (6.8%). Of these admissions, 43.1% represented a psychiatric emergency, with suicidality being the reason in 35.9%. Altogether, 72.4% had at least current passive suicidal ideation (SI, lifetime=87.2%), including passive SI (25.1%), active SI without plan (15.5%), active SI with plan (14.2%), and active SI with plan+intent (14.1%), while 11.5% had attempted suicide ≤2 weeks before admission (lifetime=28.7%). Drug-induced mental and behavioral disorders (19.6%) were the most frequent comorbid disorders, followed by personality disorders (8.2%). Upon admission, 64.5% were receiving psychiatric medications, including antidepressants (46.7%), second-generation antipsychotics (23.0%), anxiolytics (11.4%) antiepileptics (6.0%), and lithium (2.8%). Altogether, 9.8% reported nonadherence to medications within 6 months of admission.

**Conclusions:**

In adults admitted for MDD, suicidality was common, representing a psychiatric emergency in 35.9% of patients. Usual-care treatments and outcomes of suicidality in hospitalized adults with MDD require further study.

**Disclosure of Interest:**

None Declared

